# A Finite Element Model Approach to Determine the Influence of Electrode Design and Muscle Architecture on Myoelectric Signal Properties

**DOI:** 10.1371/journal.pone.0148275

**Published:** 2016-02-17

**Authors:** A. Teklemariam, E. F. Hodson-Tole, N. D. Reeves, N. P. Costen, G. Cooper

**Affiliations:** 1 School of Engineering, Manchester Metropolitan University, Manchester, United Kingdom; 2 School of Healthcare Science, Manchester Metropolitan University, Manchester, United Kingdom; 3 School of Computing, Mathematics and Digital Technology, Manchester Metropolitan University, Manchester, United Kingdom; 4 School of Mechanical, Aerospace & Civil Engineering, University of Manchester, Manchester, United Kingdom; Shanghai Jiao Tong University, CHINA

## Abstract

**Introduction:**

Surface electromyography (sEMG) is the measurement of the electrical activity of the skeletal muscle tissue detected at the skin’s surface. Typically, a bipolar electrode configuration is used. Most muscles have pennate and/or curved fibres, meaning it is not always feasible to align the bipolar electrodes along the fibres direction. Hence, there is a need to explore how different electrode designs can affect sEMG measurements.

**Method:**

A three layer finite element (skin, fat, muscle) muscle model was used to explore different electrode designs. The implemented model used as source signal an experimentally recorded intramuscular EMG taken from the biceps brachii muscle of one healthy male. A wavelet based intensity analysis of the simulated sEMG signal was performed to analyze the power of the signal in the time and frequency domain.

**Results:**

The model showed muscle tissue causing a bandwidth reduction (to 20-92- Hz). The inter-electrode distance (IED) and the electrode orientation relative to the fibres affected the total power but not the frequency filtering response. The effect of significant misalignment between the electrodes and the fibres (60°- 90°) could be reduced by increasing the IED (25–30 mm), which attenuates signal cancellation. When modelling pennated fibres, the muscle tissue started to act as a low pass filter. The effect of different IED seems to be enhanced in the pennated model, while the filtering response is changed considerably only when the electrodes are close to the signal termination within the model. For pennation angle greater than 20°, more than 50% of the source signal was attenuated, which can be compensated by increasing the IED to 25 mm.

**Conclusion:**

Differences in tissue filtering properties, shown in our model, indicates that different electrode designs should be considered for muscle with different geometric properties (i.e. pennated muscles).

## Introduction

**Fig 1:**
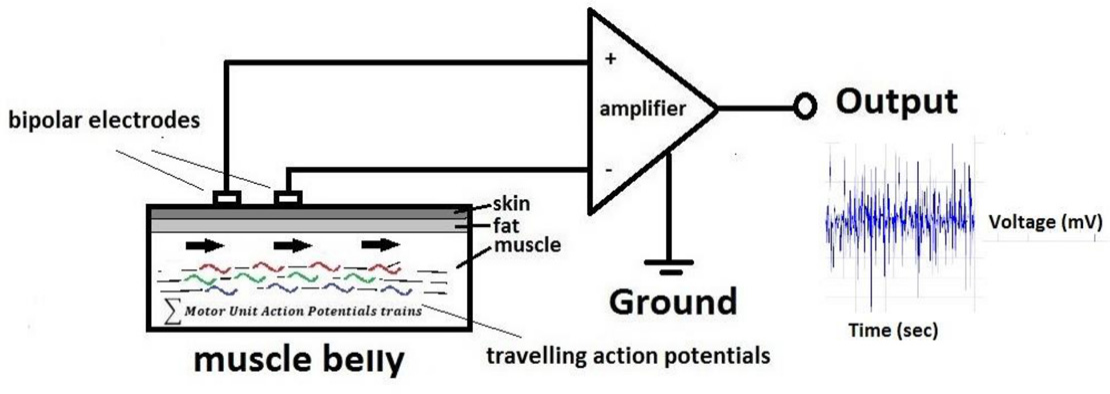
A typical Bipolar configuration scheme. Measurements are made as the difference between the signals of two sensing electrodes, separated by a known distance. The detected signal is the sum of the action potentials (MUAPs) travelling along the fibres.

A key component of the neuromuscular system is the motor unit, which is the link between neural activity and the production of muscle force. A motor unit is defined as an α-motor neuron, its axon and all the muscle fibres it innervates [1]. The motor unit action potential (MUAP) is the sum of the action potentials travelling along its fibres [2, 3]. It is possible to detect the interference pattern of these potentials using electromyography (EMG). Surface EMG (sEMG) is the measurement of the electric activity (i.e. MUAPs) detected at the skin’s surface (passing through the muscle, fat and skin), and is a good non-invasive method to estimate the level of muscle activation of superficial muscles. In order to be reliable, the measurement should: i) be representative of the muscle activity; ii) select the desired signal with minimal contribution from other signal sources (e.g. other adjacent muscles); iii) have a high precision (i.e. low error variance) and iv) have a high signal to noise ratio (SNR). To increase the SNR a bipolar electrode configuration is usually used (Fig 1), negating common node noise, but also resulting in an attenuated signal.

An important feature of the bipolar configuration is the electrode orientation with respect to the muscle fibre direction; since both sensors are supposed to see the same action potentials as they propagate along the fibre (see Fig 1). Few skeletal muscles demonstrate such parallel fibre arrangements. In contrast, most muscles have pennate (obliquely orientated) and/or curved fibres [4, 5], meaning it is not always feasible to accurately align the electrodes along the long axis of the fibres. This means that the sensing electrodes may detect signals across different fibres and the potential detected at one electrode is not the same delayed signal detected at the other electrode. This will not allow derivation of the real frequency components of the action potentials travelling along the fibre or determination of the true activation level. Therefore, there is a need to explore how different electrode orientations and distances can affect the properties of recorded myoelectric signals.

One method of investigating the effects of electrode properties on recorded signals is to employ mathematical modelling approaches. An early implementation of an analytical model of skeletal muscle tissue was reported by Andreassen and Rosenfalck [6]. The model was used to investigate the intramuscular EMG electrodes’ bipolar configuration, i.e., the orientation of the recording surfaces relative to the direction of the muscle fibres, the distance between the recording surfaces, and the area of the recording surfaces. The model parameters were the radius of the fibre; conduction velocity; anisotropy ratio of muscle tissue (difference in conductivity parallel to the fibre direction and perpendicular to it); and conductivity of the intracellular and extracellular medium. This model was able to give quantitative guidelines on the best feature set for the electrodes to provide the highest selectivity. Results suggested that a bipolar configuration with electrodes oriented perpendicular to the fibre direction gives the highest selectivity, thus recording the activity of a few fibres. This behaviour could still be considered similar if the electrode orientation angle was less than 45°. An inter-electrode center distance (IED) less than 50 μm still recorded activity in a few fibres, while IED distance greater than 200 μm gave measurements similar to that of two monopolar electrodes. This was shown by the fact that the potential amplitude decline was three times greater for the bipolar than the monopolar configuration when the fibre distance was at 200 μm, raising to five times at distances of 500 μm. This means the contribution from far fibres to the detected signal, is less in the bipolar configuration.

Merletti et al., [7] modified the Andreassen and Rosenfalck [6] action potential model. They introduced a current distribution flow from the travelling transmembrane action potential to study the effect of the innervation and termination zones on the surface signal in a parallel fibred muscle. The model was applied and compared against experimental results from sEMG signals detected from the biceps brachii muscle [8] and the model parameters set so that the model results would match the experimentally recorded signal pattern. This method gave important physiological information, for example the conduction velocity of the MUAPs and the location of the innervation zone, and provided important conclusions about the best location for electrodes, with reliable bipolar measurements possible between the innervation and termination zones, when they were sufficiently wider than the IED. However, the main limitation of this work was that the adipose tissue layer between the muscle and the electrode surface was not considered. Later Blok et al., [9] developed an analytical cylindrical volume conductor model which included the skin and fat layers to describe the muscle tissue, improving previous two layers models, which were sensitive to the depth of the potential source [10]. Wheeler et al., [11] extended these models by defining and modelling the motor units size, properties (e.g. conduction velocity), validating the simulated sEMG, and derived force by experimental observation. They reported a strong correlation between linear sEMG and force relationships found in both simulated and experimentally recorded signals. While these models have provided valuable guidelines, wider applications are limited by the fact that analytical models can only provide solutions for ideal geometries such as infinite cylinders or planar planes and symmetric geometries [12]. The potential to investigate properties of more realistic muscle geometric properties can however be done using a finite element model (FEM) approach.

FE modelling is a mathematical method that discretises a continual space into finite elements, turning differential equations into algebraic equations for which it is possible to find an approximate solution. FEMs therefore extend the possible range of studies, specifically in terms of the complexity of the muscle fibre geometries that can be considered. For example, Lowery et al. [13] reported an FEM of a cylindrical three-layered muscle tissue which was further implemented into an anatomically based (real muscle geometry) model which included the bone and blood vessels, and showed how these additional components can change the amplitude of the EMG signal and the shape of the action potential. Pennated and curved fibres can change the action potential shape detected, and hence the frequency components of the EMG. This was shown by Mesin et al. [14] who studied conductivity property changes during shortening of a 3D fusiform shaped FEM of the muscle and, in agreement with previous analytical models [14], revealed changes in amplitude and frequency content of the action potential.

Previous FEM all make use of single MUAP trains, which makes it harder to validate against commonly recorded myoelectric signals, which represent the interference pattern of multiple MUAPs. In the present study, a different approach was used. The fibres of a motor unit cover a portion of the cross section of a muscle with a round irregular shape [15, 16] and the activity of a small number of fibres at a certain depth can be detected through intramuscular EMG (iEMG). Instead of modelling the current of a single fibre, the potential detected can be considered as the superimposed resultant potential at that depth, hence the source signal is the overall potential travelling at that depth, which can be detected through intramuscular measurements. Using this approach, it is possible to implement models that use a source signal acquired from experimental acquisition and is thus closer to the real signal than simulated data. This approach enables greater focus on the filtering properties of the muscle tissues and the surface electrodes configuration with respect to the fibres. The aim of this work was to simulate the muscle tissue volume conductive properties and test the effect of inter-electrode distance, electrode orientation and muscle fibre pennation angle on the simulated bipolar sEMG signal. Differences in simulated sEMG signals were explored in terms of power loss and frequency filtering. This aim was pursued by implementing a three layer FEM of the muscle, fat and skin.

## Method

### FE Model

**Fig 2:**
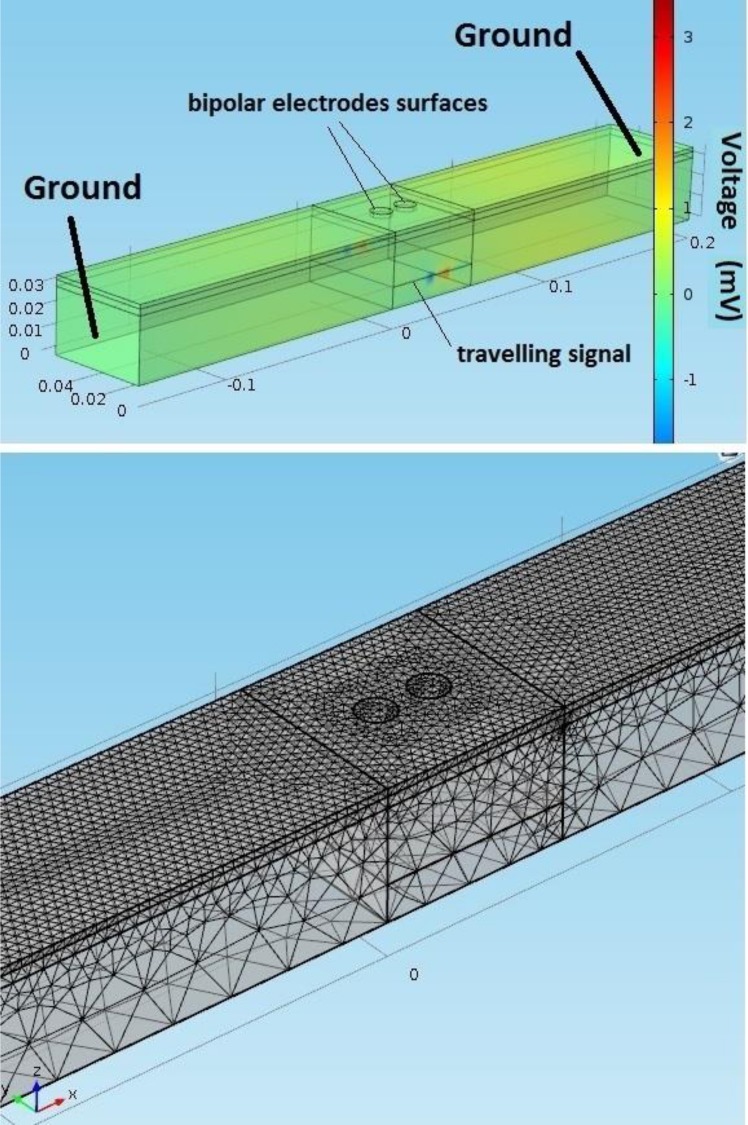
**(Left) Three layers FEM of the muscle tissue.** The simulated model consisted of an intramuscular travelling potential which generated a potential on the surface of the model. The surface potential is then detected by two probe areas representing the electrodes. These probes record the average potential over the area, reflecting the behaviour of sEMG electrodes. The coloured bar represents the tissue electric potential. **(Right) FEM four node tetrahedral mesh.** A finer mesh was built in the electrodes areas.

A three layer FEM model of the muscle (2 mm skin, 3 mm fat, 30 mm muscle, see Fig 2) was implemented in the time domain [17] using Comsol Multiphysics (version 4.4, Cambridge, UK) explicit finite element solver. A conjugate gradient iterative solver was used. A convergence h-refinement study was also performed for 6 levels of mesh density by refining the mesh globally from 3462 to 87353 elements. The parameter chosen to measure convergence was the root mean square of the signal, at the electrodes since it represents the signal total energy. The results converge to around 2.17 × 10^−6^ mV and 2.12 × 10^−6^ mV for the first and second electrodes. The mesh design of our FE model used 51053 solid tetrahedral elements.

The model set up was similar to that proposed by Lowery [12, 13, 18]. Specifically our model used a quasi-static assumption applied to the biological tissues [19], hence the muscle can be considered as a resistive tissue only (ignored effects from capacitance and inductance). The governing equation is a combined form of the continuity equation and Gauss’s law in the differential form [20]:

∇⋅D=ρ(Gauss Law) (1)

$$\nabla \cdot \text{J}=\frac{\partial \rho }{\partial \text{t}}\;\left(\text{Charge continuity equation}\right)$$ (2)

ρ=charge densityD=electric displacementJ=current density

Ohm’s law and the constitutive equation are used to relate the electric field with the electric displacement and the current density:

$$\text{D}=\varepsilon_{0}\varepsilon_{\text{r}}\text{E}$$ (3)

J=σE (4)

$$\begin{array}{l} \text{E}=\text{electric field}\;\varepsilon_{0}=\text{vacuum permittivity}\;\varepsilon_{\text{r}}=\text{relative permittivity}\\ \sigma =\mathrm{electrical conductivity} \end{array}$$

The skin and fat tissue were considered as isotropic while the muscle anisotropy ratio (longitudinal conductivity over transversal conductivity) was set to 5 [12, 21]. Table 1 reports the dielectric properties used in the model. The dielectric properties were taken at a frequency of 100 Hz which is the typical median frequency of an EMG spectrum [12].

**Table 1:** Dielettric properties of the muscle tissues [22].

	Conductivity (S/m)	Permittivity
**Muscle**	0.26671*	9329000
**Skin**	0.00046112	45298
**Fat**	0.02081	457060

*tranverse

Combining Gauss’s law and the continuity equation, by eliminating the charge density a single equation is obtained:

$$\nabla \cdot (\sigma \text{E}+\frac{\partial \varepsilon \text{E}}{\partial \text{t}})=\;0$$ (5)

In a quasi-static approximation the electric potential (V) is given as:

E=−∇V (6)

Substituting Eq 6 into Eq 5:

$$-\nabla \cdot (\sigma \nabla \;\text{V}+\frac{\partial \varepsilon \nabla \;\text{V}}{\partial \text{t}})=\;0$$ (7)

Eq 7 is the governing equation for time-dependent electric currents.

Boundary conditions are specified at material interfaces and physical boundaries. At the interface between two media (skin, fat, muscle), the Neumann boundary condition is given for the current density:

$${\text{n}}_{2}\cdot ({\text{J}}_{1}-{\text{J}}_{2})=-\frac{\partial \rho }{\partial \text{t}}$$ (8)

Where n_2_ is the outward normal from medium two. In a resistive media, the current density should be continuous:

$${\text{n}}_{2}\cdot ({\text{J}}_{1}-{\text{J}}_{2})=\;0$$ (9)

The portion of muscle simulated was considered electrically isolated at its boundaries since the conductivity of the surrounding space (air) can be assumed to be zero; therefore, there is no normal current flow:

n⋅J=0 (10)

The ground was applied at the extremities of the model, where the electric potential V is null (Dirichlet boundary condition).

### Input Data

The model simulates the whole intramuscular MUAP interference pattern and evaluates the surface potentials at two areas representing sEMG electrodes. To simulate the MUAP signal, the implemented model used a recorded iEMG signal (sample frequency 5 KHz, amplifier gain 1000) from fine-wire electrodes (3 mm bared tips) inserted in the long head of the biceps brachii muscle (see data in S1 Datafile). The measurements were taken from one healthy male, adult volunteer. The participant provided written informed consent and the study was approved by the local ethics committee at the Faculty of Science and Engineering, Manchester Metropolitan University, in accordance with principles of *Declaration of Helsinki*. The iEMG was recorded at a 20 mm depth during a 10% maximum voluntary isometric contraction (a static model). It was assumed that this signal was representative of the intermuscular EMG across the whole muscle volume. A 50Hz notch filtered 1 sec portion of the iEMG was applied as the source signal in the simulation. This source signal was set as an electric potential *V*(*t*) that travelled for one second along a surface within the muscle domain:

$$V\left(t\right)=signal\;\left(t-\frac{x}{v}\right)$$ (11)

Where *x* is the coordinate along the fibre direction, and *v* is the speed of propagation (4 m s^-1^). The source signal travels along a distance of 0.05 m while the tissue extends to 0.15 m at both sides (see Fig 2), a distance by which the potential is no longer affected by the boundaries of the model. To define this distance a correlation study was conducted to assess the minimum extension length for which the outcome distance is not affected by the drop of the electric field at the end of the signal route (see Table 2).

**Table 2:** Correlation between the RMS of the resultant bipolar potentials at different muscle lengths (extension). After a length of 100 mm, the results are strongly correlated.

Length (mm)	0	50	100	150	200	250	300
**0**	1	0.66751	0.567246	0.552953	0.542169	0.5422	0.546311
**50**	0.66751	1	0.837897	0.84586	0.850144	0.848236	0.853335
**100**	0.567246	0.837897	1	0.998694	0.997279	0.996393	0.997045
**150**	0.552953	0.84586	0.998694	1	0.999275	0.998708	0.999119
**200**	0.542169	0.850144	0.997279	0.999275	1	0.999665	0.999854
**250**	0.5422	0.848236	0.996393	0.998708	0.999665	1	0.999595
**300**	0.546311	0.853335	0.997045	0.999119	0.999854	0.999595	1

**Fig 3:**
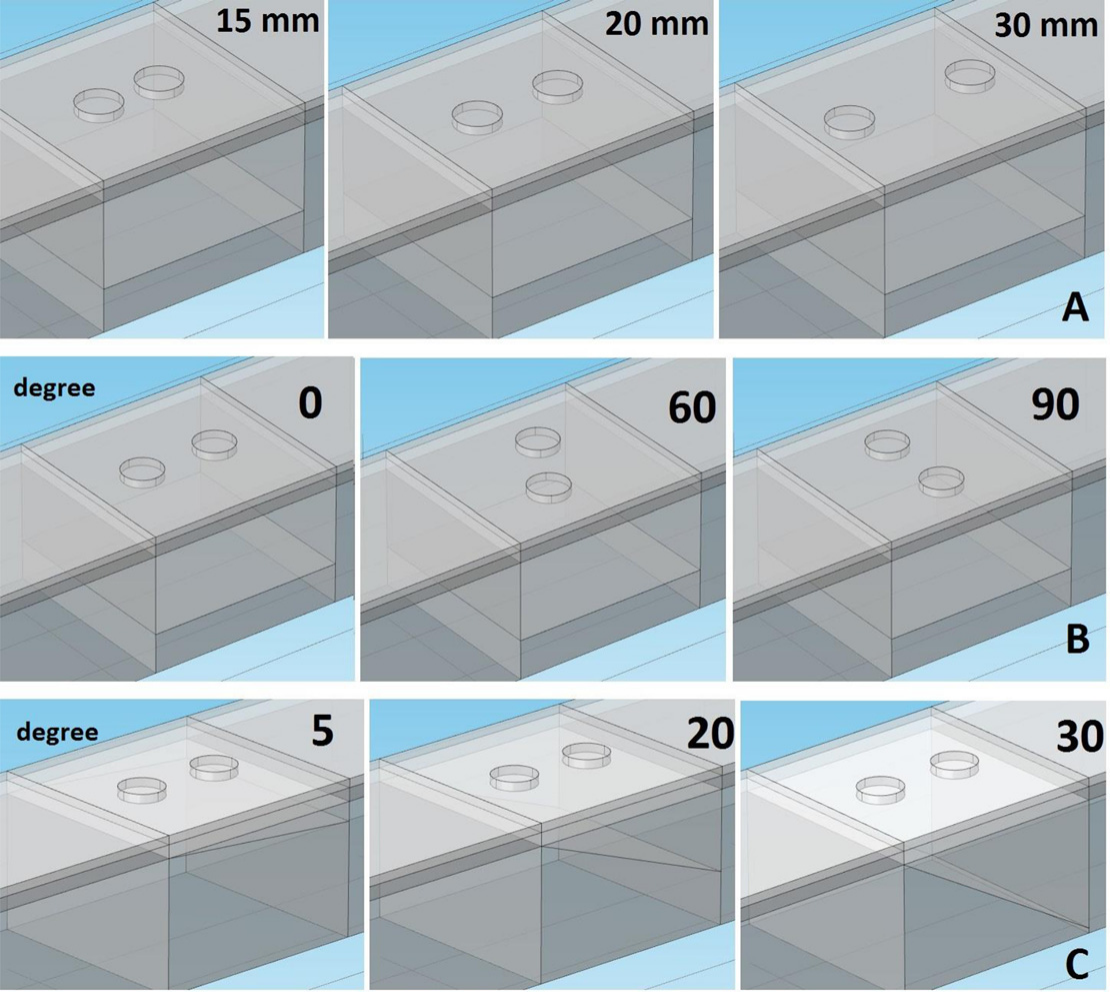
Representations of some of the bipolar configurations simulated, showing:. A) inter-electrode distances (mm); B) electrodes orientation (Degree); C) fibre pennation angle (Degree). The signal travels along the horizontal plane in the muscle tissue at a depth of 20 mm. For the pennated case, the plane is inclined at different angles.

Two probe circular areas (radius, 50 mm) on the skin represent the electrodes (see Fig 2). The average potential on these areas were recorded and post processed. A bipolar electrode configuration was investigated by changing three different parameters: the IED, the orientation of the electrodes and the pennation angle of the fibre along which the signal travelled (see Fig 3). The inter-electrode distance was set at 15, 20, 25, 30 mm; the orientation of the electrodes was varied to 0°, 10°, 30°, 60°, 90° and the fibre pennation angle changed to 5°, 10°, 15°, 20°, 30°. In the parallel case the signal travels at 20 mm depth from the surface.

### Signal Processing

**Fig 4:**
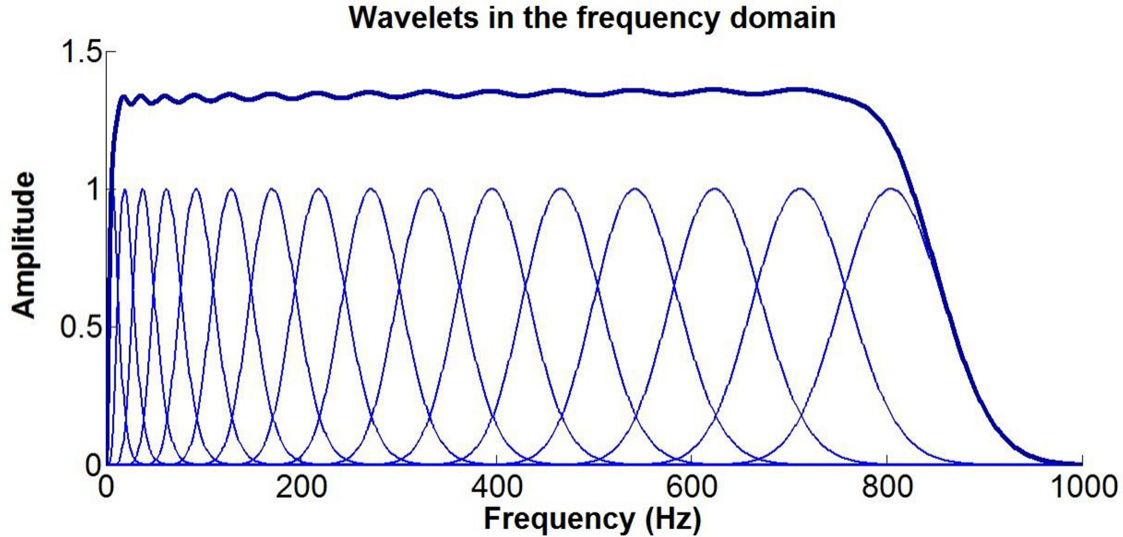
The 16 wavelets set in the frequency domain. The sum of the wavelet (thick line) gives the total band of the filter. Each peak is the central frequency of the single wavelet.

Matlab (version r2013a, Cambridge, UK) was used for post processing. A wavelet based intensity analysis of the simulated sEMG signal was performed [23]. This method uses a filter bank of non-linearly scaled wavelets developed specifically for EMG time frequency analysis, with applications to both experimental and simulated signals described in detail elsewhere [24–26]. Briefly, based on the source signal sample frequency (5000 Hz), a set of 16 wavelets with center frequencies spanning 6.9Hz to 804Hz (see Eq 5) was defined in the frequency domain (see Fig 4).

$$F\Psi \left(f,cf,scale\right)≔{\left(\frac{f}{cf}\right)}^{cf\cdot scale}\cdot e^{\left(\frac{-f}{cf}+1\right)\cdot cf\cdot scale}$$ (12)

The EMG signal is then convolved with the wavelet set, essentially band-pass filtering the signal with the same frequency components as the wavelets [25]. Using this method, an intensity can be calculated which approximates the power of the signal [27]. This method enables investigation of the filtering effects (power attenuation) of electrode configuration and fibre geometries at the central frequencies of the wavelet analysis.

The mean power of the sEMG bipolar signal, expressed as a percentage of the mean power of the iEMG (source) signal was used to interpret the results.

## Results

### Properties of the iEMG Source Signal

**Fig 5:**
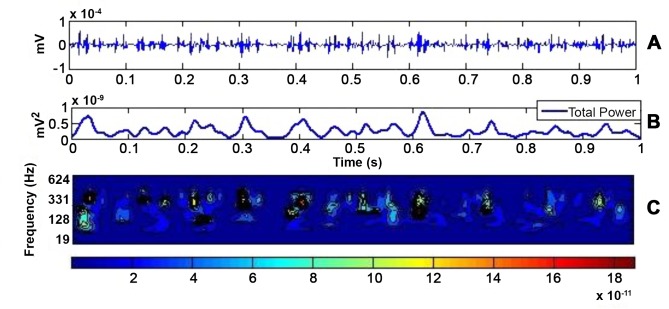
The source signal power in time and frequency domain. The 1 sec sample from intramuscular signal that was used as source signal is shown at the top (A). The total power of this signal is shown in the time domain (B) and in the frequency-time domain (C). From this plot it is possible to distinguish bursts of activity that occur during the isometric contraction. These are represented by high frequency peaks in the time domain, amplitude peaks in the power plot and red yellow region in the time-frequency domain. It can be seen that the power peaks are mainly at higher frequencies.

The source signal contained bursts of activity at short time intervals, which indicate the activation of recorded motor units (see Fig 5A). Typically, the bursts occur at higher frequencies (indicating appropriate ranges), and are evident in the total power plot (Fig 5B) as amplitude peaks. From the time-central frequency power plot (Fig 5C) it is also evident that lower frequencies (<62 Hz) contribute minimally to the recorded signal.

**Fig 6:**
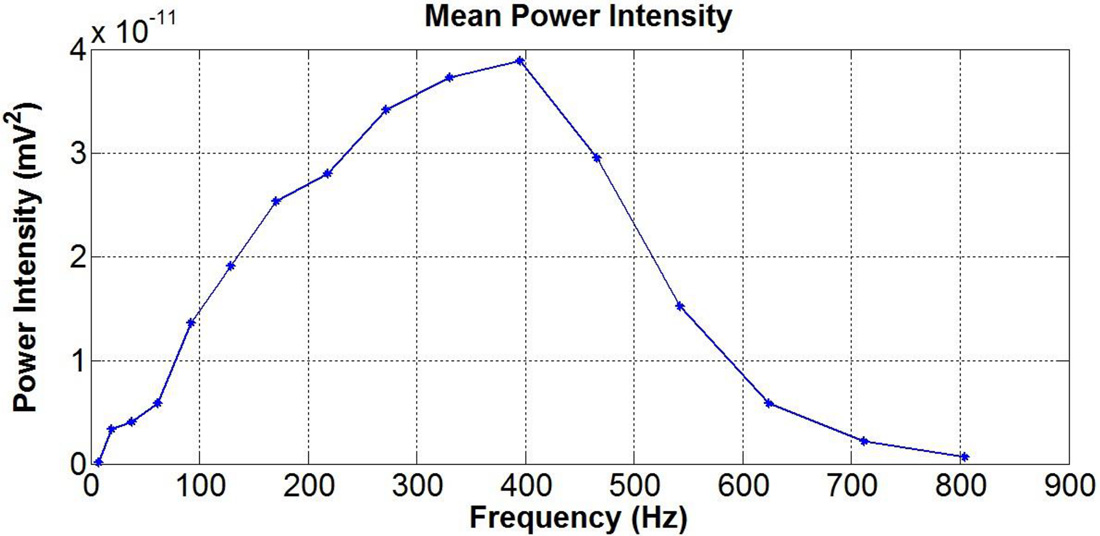
The source signal mean power. The source signal mean power over 1 sec calculated for each frequency component of the source signal.

In Fig 6 the source signal mean power for each central frequency is reported. The mean power of the intramuscular source signal had peak values around the central frequency of 395 Hz.

### Influence of Inter-Electrode Distance & Orientation

**Fig 7:**
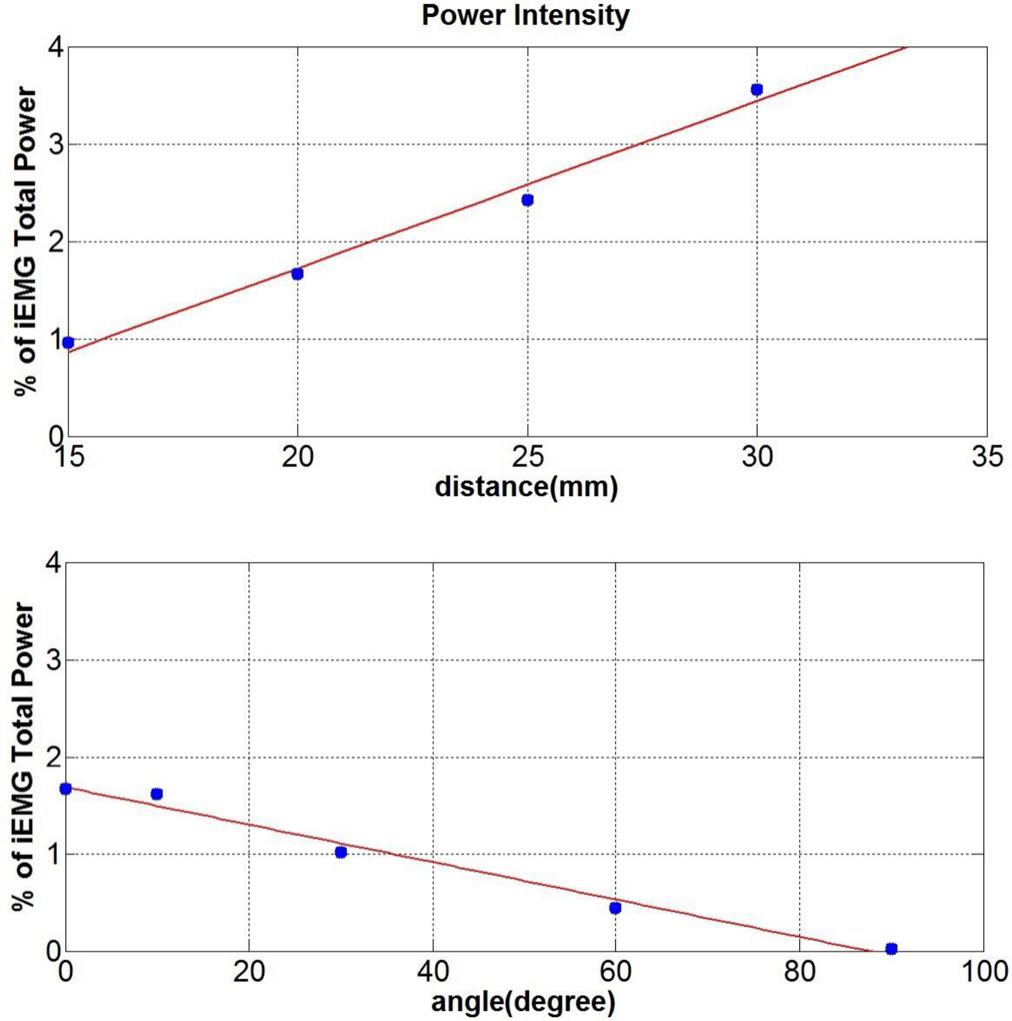
Total Power expressed as percentage of the source signal total power. (Top) Total power for different IED, showing a positive linear trend as the distance is increasing (Bottom) Total Power at different orientation, showing a decay as the electrodes alignment deviate from 0° to 90°.

The overall energy of the signal was assessed by evaluating the total power measured at the simulated surface electrode. A positive linear relationship was found between the IED and the total power, while a negative decay was found as the electrode alignment deviated from the signal direction toward a perpendicular orientation (see Fig 7). The difference in the total power as a percentage of the source signal power between the lowest and the highest parameter was just 3% when varying the IED, and around 2% when the electrode orientation was varied.

**Fig 8:**
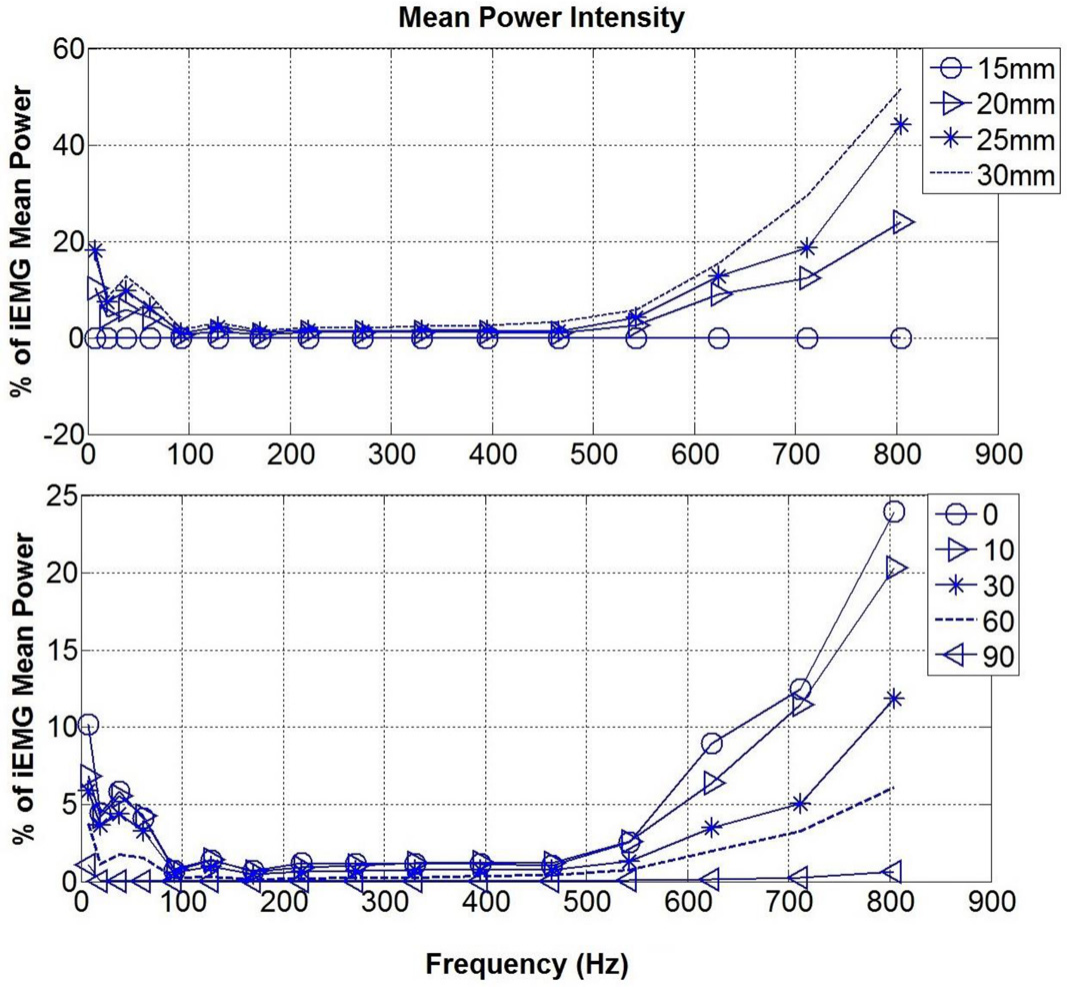
Mean Power of the bipolar signal in the frequency domain. The general trend reveal a band stop filtering in the range 92–542 Hz (Top) Mean power at different IED. As the distance increases, the mean power increases as well. (Bottom) Mean power at different orientations. As the electrodes deviate from the direction of the signal, the mean power decreases, until reaching almost zero at 90°.

The mean power of the bipolar surface signal for each central frequency was also obtained (see Fig 8). The mean power increased as the IED was increased, but this occurred only for lower (<92 Hz) and higher (>542 Hz) frequencies, with the same effect occurring at these frequency ranges for the different electrode orientations investigated.

### Influence of Fibre Pennation Angle

**Fig 9:**
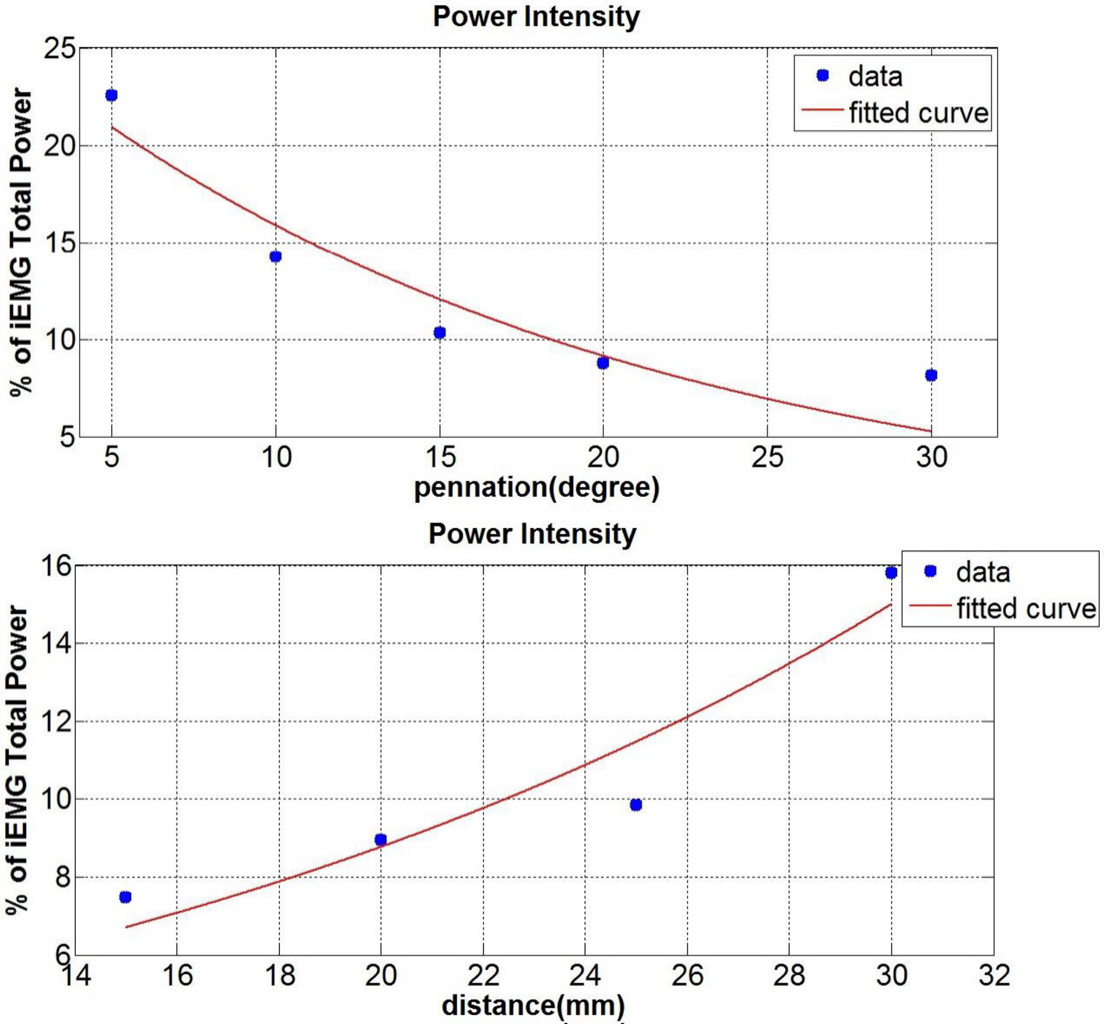
Total Power of the bipolar configuration. (Top) Total power at different fibre pennation angles. There is a linear decrease of the power as the pennation increase for a fixed IED of 20 mm. (Bottom) Total Power for a pennated model at 20° while changing the IED. The increase in the power as the distance increase is almost double as that found in the parallel fibres model.

The amplitude of the sEMG from a signal travelling along a simulated pennated muscle fibre was also increased and of the same order of amplitude as the source signal. As the pennation angle increased the total power of the bipolar signal was considerably attenuated (up to 13%, Fig 9 top). Combining the effect of changing the IED with a 20 ^o^ pennation angle showed an enhanced effect on the total power (see Fig 9 bottom). A 7% difference in the total power between the lowest and the highest IED case was found, while it was just of 3% without pennation.

**Fig 10:**
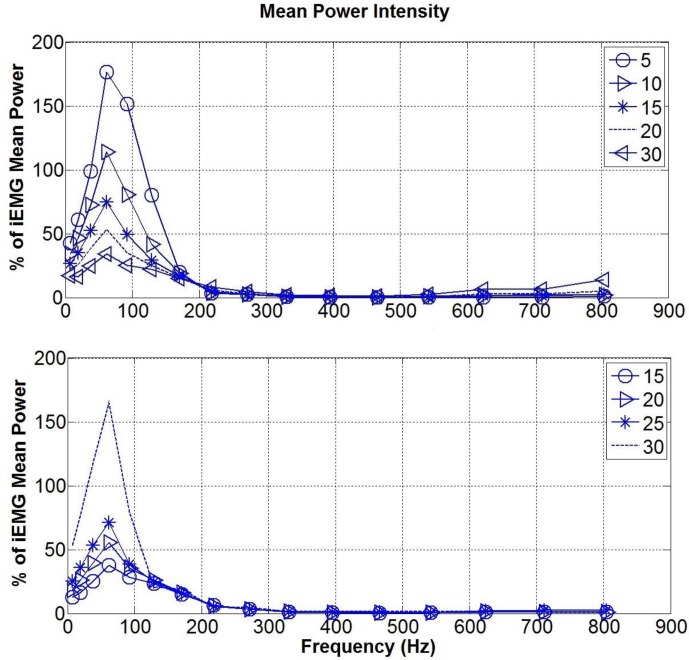
Mean Power of the bipolar signal in the pennated case. (Top) The mean power at different pennation is low pass filtered and the amplitude increase as the pennation decrease. (Bottom) Mean power for the pennated case at 20° while changing the IED (15,20,25,30 mm). There is an enhanced peak for the IED of 30 mm for which the electrodes are close to the terminating point.

The fibre pennation angle strongly affected the outcome signal, which was low pass filtered, retaining frequencies lower than the central frequency of 170 Hz (see Fig 10 top). The IED did not seem to affect the filtering response except when the electrode was relatively close to the termination side of the signal (see Fig 10 bottom). In that case (IED 30 mm) a narrower band and prominent peak at the central frequency of 62 Hz was found.

## Discussion

A FEM of the muscle tissue was simulated to study the performance of different sEMG electrodes in bipolar configurations. During experimental data collection, the configuration of the electrodes is important to avoid cross-talk [28] and to assess the detection volume [29]. Bipolar measurements are more selective since they show a lower detection range [6], but are sensitive to their orientation with respect to the direction of the muscle fibres and the distance between the two electrodes [6].

### Orientation and IED

From the work presented here, it is clear that the orientation of the electrode areas should give a differential signal similar to that of the IED, since the signal considered in the first two simulations is travelling horizontally across the tissue layer. As the electrodes become misaligned with respect to the signal direction, the signal detected will give a bipolar measurement equivalent to that from electrodes that have a smaller inter-electrode distance than that of the actual electrodes (considering an area across which MUAPs are homogeneous). The case in which the electrode areas are aligned at 90° with respect to the signal direction can be associated with the case of a null inter-electrode distance, hence the differential measurements cancel out. Andreassen and Rosenfalck [6] concluded that for an angle above 45°(less than 45° according to their reference system), the iEMG electrodes will act as a perpendicular oriented electrode pair. In the simulations performed here the IED and the electrode orientation seems to play a minor role when the signal is travelling along a plane parallel to the electrode plane. The influence of these features is to change the power amplitude while leaving the tissue filtering properties unchanged. Fig 8 shows that the main attenuation of the signal intensity occurs between 92–542 Hz, which is a range that includes frequencies of physiological relevance. Slower motor units operate in the frequency range between 82–247 Hz, while faster in the range 240–423 Hz [26, 30]. Higher frequencies (>542 Hz) are less attenuated and the effect of different IED and electrode orientation is more remarkable, but these frequencies are generally discarded by researchers, as they are not in the physiologically relevant frequency range. This could be said to be a bandwidth reduction similar to that observed by Lindstrom et al [31]. When the misalignment (see Fig 8 bottom) between the electrodes and the fibres is significant (60°- 90°) it is suitable to increase the IED distance (25–30 mm) to reduce the chance of the signal cancelling out in a bipolar configuration. By choosing the IED, other features considered in previous models should be also evaluated. The IED should not be too large in order to avoid the termination end effects [7]. Moreover, the thickness of the fat tissue increases substantially the pick-up volume, which is further extended by the IED [18]. This can lead to the occurrence of cross-talk [32] with other muscle activities.

### Pennation

When dealing with pennated fibres, the signal travels along a diagonal plane. This fact changes the filtering response of the muscle tissue model. While the muscle tissue acted as a band stop filter (attenuating the wavelet frequency range between 92–542 Hz) when parallel fibres were modelled, it started to act as a low pass filter when pennation was considered (Fig 10). This change in the frequency response can be explained as a consequence of the bipolar configuration, with the higher contribution to the sEMG signal coming from the electrode which is closer to the source signal [33] and changes in the detected potential shape as occurring as predicted by the analytical pennated model proposed by Mesin and Farina [34]. The effect of a different IED seems to be enhanced in the pennated model in terms of total power, while the filtering response is changed considerably only for IED where the electrodes are close to the signal termination points (IED 30 mm). In addition, the amplitude of the sEMG is generally increased in the pennated simulation due to the small distance between the signal and the muscle surface as it starts to travel toward the deep aponeurosis. For the isometric condition studied here, Fig 10 suggests that for pennation angles greater than 20° (more than 50% of the source signal is attenuated) it is suitable to increase the IED, i.e. IED of 20–25 mm. In our simulation, the signal is the sum of the MUAPs, hence a simplification was made in considering it travelling from the top to the bottom toward the deep aponeurosis. Muscle fibre’s motor units end plate are usually located in the middle of the fibres [35]. This implies that in pennated fibres, the single action potentials travel in both directions toward the deep and the superficial aponeurosis. Nevertheless the direction of the potential detected, depends on the relative position between the bipolar electrodes, the fibres and the motor unit end plate [36]. Therefore, the source signal could have been represented in different ways, which might depend on the muscle that is being studied and its pennation. One major feature that this model predicted is the fact that the potential depends on the signal arriving at the superficial aponeurosis (closer to the electrodes), while the signal ending at the deep aponeurosis is strongly attenuated. This make it possible for signals to be recorded from different groups of muscle fibres and provide spatially localized information [37].The geometry of the fibres can also change as the muscle is contracting as addressed in Mesin et al. [14] analytical model. Despite the limitation of not including the skin and connective tissue, they showed the importance of considering dynamic changes, specifically for recording the activity of deeper fibres, which are more affected as the distance from the detecting electrode is changed [15]. They also showed that the shortening of the muscle influenced the weight of the non-propagating components (standing waves) in the signal. Therefore, the model presented in this paper can only provide insight into the effects of electrode configuration or pennation during isometric contractions and further work is required to enable investigation of more dynamic contractions.

## Conclusion

From this study it can be seen that sEMG electrode configurations become an important feature when dealing with pennated muscles. This is because the signal travelling along a diagonal plane will be filtered differently by the electrodes in a bipolar configuration and will be attenuated while travelling down. An experimental approach to overcome this problem, using current measurements instead of potentials has been proposed, with results suggesting an increase in the spatial resolution [32, 33]. During most functional motor tasks there will be changes in fascicle pennation angle as a result of muscle activation and so this is a dynamically changing feature of the muscle, which will influence what the electrodes record. For this reason, it is important to take these features into consideration when designing EMG electrodes. To provide a foundation from which future guidelines may be set, future work should investigate the effect of dynamic changes in fascicle geometry and include consideration of other anatomical features such as tissue curvature (i.e. skin surface and fascicle) and skeletal elements.

The model presented here is able to evaluate the combined effects of electrode configuration and muscle architecture; it can be used to provide insight into the impact of changes in muscle pennation angle, on sEMG activity measured [14]. The model in this study focused on a rectangular geometry with straight muscle fibres. Future work will include the study of fibre end effects on the detected signal, to explore the phenomenon of standing waves that occur as the signal reaches the termination of the fibres. Another possible application of this model involves errors in the estimation of the conduction velocity when the electrodes are not correctly aligned with the fibres direction. Misalignment between the electrode and the fibres leads to a lower estimation of the conduction velocity, since the conduction velocity is estimated through the delay that a certain signal pattern takes when travelling between the pick-up area of an electrode pair [8].

It is recognised that this model only activates a small region of muscle but in practice, real activations may occur across regions of muscle[38], which have different sizes or geometry. Further work could use medical imaging such as ultrasound to quantify active regions in the muscle which could then be used to provide more accurate estimations of the active muscle region [39].

## Supporting Information

S1 Datafile Experimental source data. This file contains the data set of the experimental iEMG used in the simulation as source signal.
(TXT)
